# Nanoengineered, Pd-doped Co@C nanoparticles as an effective electrocatalyst for OER in alkaline seawater electrolysis

**DOI:** 10.1038/s41598-023-46292-9

**Published:** 2023-11-27

**Authors:** Zafar Khan Ghouri, David James Hughes, Khalid Ahmed, Khaled Elsaid, Mohamed Mahmoud Nasef, Ahmed Badreldin, Ahmed Abdel-Wahab

**Affiliations:** 1 School of Computing, Engineering and Digital Technologies, Teesside University, Tees Valley, Middlesbrough, TS1 3BX UK; 2 Center of Hydrogen Energy, Institute of Future Energy, Universiti Teknologi Malaysia, Jalan Sultan Yahya Petra, 54100 Kuala Lumpur, Malaysia; 3 International Center for Chemical and Biological Sciences, HEJ Research Institute of Chemistry, University of Karachi, Karachi, 75270 Pakistan; 4 Chemical Engineering Program, Texas A&M University at Qatar, P.O. 23874, Doha, Qatar; 5 Malaysia-Japan International Institute of Technology, Universiti Teknologi Malaysia, Jalan Sultan Yahya Petra, 54100 Kuala Lumpur, Malaysia

**Keywords:** Chemistry, Materials science, Nanoscience and technology

## Abstract

Water electrolysis is considered one of the major sources of green hydrogen as the fuel of the future. However, due to limited freshwater resources, more interest has been geared toward seawater electrolysis for hydrogen production. The development of effective and selective electrocatalysts from earth-abundant elements for oxygen evolution reaction (OER) as the bottleneck for seawater electrolysis is highly desirable. This work introduces novel Pd-doped Co nanoparticles encapsulated in graphite carbon shell electrode (Pd-doped CoNPs@C shell) as a highly active OER electrocatalyst towards alkaline seawater oxidation, which outperforms the state-of-the-art catalyst, RuO_2_. Significantly, Pd-doped CoNPs@C shell electrode exhibiting low OER overpotential of ≈213, ≈372, and ≈ 429 mV at 10, 50, and 100 mA/cm^2^, respectively together with a small Tafel slope of ≈ 120 mV/dec than pure Co@C and Pd@C electrode in alkaline seawater media. The high catalytic activity at the aforementioned current density reveals decent selectivity, thus obviating the evolution of chloride reaction (CER), i.e., ∼490 mV, as competitive to the OER. Results indicated that Pd-doped Co nanoparticles encapsulated in graphite carbon shell (Pd-doped CoNPs@C electrode) could be a very promising candidate for seawater electrolysis.

## Introduction

The growing need for energy and concerns about fossil fuel consumption necessitates using green and sustainable energy sources^1,2^. Hydrogen, as a clean fuel, has gotten huge attention and has been thoroughly investigated as a viable alternative to fossil fuels^3,4^. Despite its tremendous application potential, current commercial hydrogen production relies on fossil fuels with huge carbon emissions^5,6^. As a result, it is critical to investigate a cleaner, more sustainable, and more efficient strategy for hydrogen production. Water splitting with high energy conversion efficiency has recently been exploited as an efficient and environmentally friendly option^7,8^. Therefore, electrochemical water splitting, i.e., water electrolysis, is currently being explored for the futuristic expansion of hydrogen production^9^. As water electrolysis has shown promising results, however, freshwater resources are scarce in many regions worldwide. Although seawater is an abundant resource, accounting for almost 97% of the planet's water, seawater electrolysis is still in its infancy^10,11^. Therefore, seawater electrolysis has become a hot research topic, which is mainly based on anodic oxygen evolution and cathodic hydrogen evolution reactions, i.e., OER and HER, respectively. Unfortunately, the presence of electrochemically active chloride anions, which may severely deteriorate the electrocatalysts and compete with the OER is considered the main obstacle to seawater electrolysis^10,11^. Furthermore, the complex chemistry of seawater might cause insoluble precipitates to develop on the active site of the catalyst surface, lowering its performance^12–15^.

Significant activation barriers in the HER and OER pathways lead to large overpotentials, resulting in slow water electrolysis kinetics^16,17^. To reduce the activation energy barrier, lower the overpotential, and eventually higher energy conversion efficiency, electrocatalysts should poses high activity^18^. Therefore, it has great importance to develop active and selective electrocatalysts to drive the OER activity in seawater environment^19^. Water electrolysis preferably take place in acidic or alkaline media. Unfortunately, the lack of efficient and low-cost catalysts makes acid electrolyzers technologically and commercially unviable^3,20^. As a result, huge efforts have been made to produce catalysts with high activity and stability in alkaline media based on existing alkaline oxygen evolution electrocatalysts, to speed the commercialization of alkaline electrolyzers for hydrogen production.

In general, noble metal-based catalysts are thought to be the best electrocatalysts for stimulating electrochemical reactions such as HER, OER, and oxygen reduction reaction (ORR)^21,22^ but they are costly and scarce, making them unsuitable for large-scale commercial production^23,24^. As a result, finding low-cost, high efficiency electrocatalysts for is paramount. This motivates a worldwide search for non-noble metal-based alternatives, such as Co-^25^, Ni-^26^, and Mo-based catalysts ^27^. Co-based catalysts, in particular, are excellent low-cost electrocatalysts for OER^4^. Various Co-based catalysts, such as CoO^28^, CoSe_2_^29^, CoP^30^, CoPS^31^, Co_2_B^32^, CuCo^33^, and FeCo^34^, have sprung due to their simple preparation, low cost, and excellent activity as well as durability when compared to other Co-based complexes^35^. Bimetallic catalysts that combine platinum group elements with Co usually provide higher catalytic performance through cooperative effect that is not visible in their monometallic counterparts^4,36^.

Theoretical and experimental data show that alloying platinum group elements with Co alter the average surface energy as well as the width of the d-band due to cumulative strain and ligand effect. This can lead to changes in surface chemical characteristics and a significant increase the activity^37,38^. Palladium, has been explored as a potential alloying element because of its stronger affinity for OER and lower cost than Pt^39^. In addition, integrating alloy with carbon substrates, especially encapsulating them in heteroatom-doped carbon, can enhance OER performance^8,37^. Furthermore, Bao et al. reported that combining a carbon shell with a metal core could boost activity and stability at the same time. Recently, N-doped graphene-encased metal or alloy has emerged as a novel and exciting electrocatalysts^40^. Furthermore, electrons would flow from the metal core to the outside carbon layer due to the distinct working functions of metal and nonmetal, increasing the density of carbon states near the Fermi level and optimizing their kinetic processes for various fundamental steps^41^. As a result, the overall activity of metal-doped carbon structures can be tailored using either the carbon shell or the metal core. Accordingly, electrical structures of carbon can be modified to boost their activities by altering doping types, doping concentrations, element types, metal proportions, and structures in the alloy core^42^. In this study, Pd-doped Co nanoparticles encapsulated in graphitic carbon shell (Pd-doped CoNPs@C electrode) are developed with simple and cost-effective route. The developed catalyst is then assessed as a selective anode towards cost-efficient OER under alkaline pH conditions from seawater splitting.

## Experimental section

### Chemicals and materials

Palladium (II) acetate PdAc (Pd (CH_3_CO_2_)_2_.), Cobalt (II) acetate CoAc (Co (CH_3_CO_2_)_2_), and Poly (Vinyl Alcohol) PVA (Mw 85,000–124,00) were purchased from Sigma-Aldrich. All the chemicals were used as received without any further purification. Milli-Q ultrapure water (≈18.2 MΩ) was used for all the syntheses.

### Synthesis of Pd-doped Co nanoparticles encapsulated in graphite carbon shell

**Figure 1:**
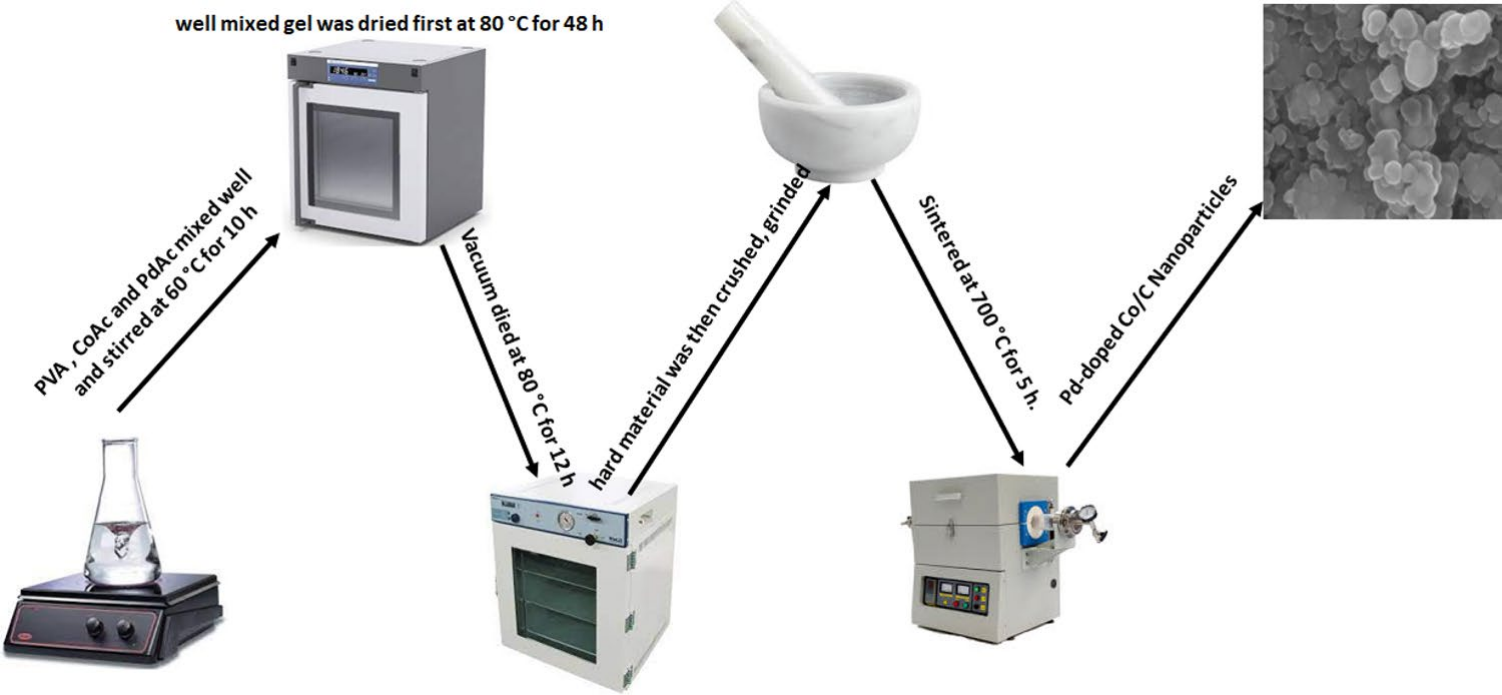
Schematic diagram for the process and final product.

A typical synthesis of Pd-doped CoNPs@C electrode was conducted as follows: 5.0 g of 10 wt% PVA, 0.8 gm of CoAc, and 0.2 gm of PdAc were mixed very well with continuous stirring at 60 °C for 10 h. The obtained so-gel was dried first at 80 °C for 48 h and then under vacuum at 80 °C for 12 h. Finally, the obtained solid mixture was crushed, ground, and sintered under an Ar atmosphere at 1 atm and 700 °C for 5 h. Figure 1 shows the schematic of the synthesis procedure for Pd-doped CoNPs@C electrode.

### Characterizations

Field-emission scanning electron FESEM-FEI microscopy (ThermoFisher Scientific, MA, USA), transmission electron (TEM) microscope (JEM-2100F, JEOL, Japan), and high-resolution TEM (HRTEM) techniques were utilized for morphological characterizations while, the X-ray diffractometer XRD (XRD- D/max 2500 Rigaku, TX, USA) and X-ray photoelectron spectroscopy XPS (AXIS Ultra DLD, Kratus, UK) techniques were used to analyze the phase structure/crystallinity and the surface chemistries of the prepared Pd-doped CoNPs@C electrode, respectively. The element loading amount was estimated by Inductively coupled plasma mass spectrometry ICP-MS (Perkin Elmer NexION 300D).

### Electrochemical measurements

Electrochemical tests were performed using Reference 3000 electrochemical workstation (Gamry, PA, USA). The catalytic performance was evaluated in 1.0 M KOH and 1.0 M KOH + 0.6 M NaCl aqueous solution with a conventional three-electrode system. The Ag/AgCl electrode and Pt wire were used as reference and counter electrodes, respectively. The glassy carbon as a working electrode was prepared with 15 µL of the catalyst ink composed of 2.0 mg of catalyst nanoparticles, 200 µL n-propanol, 200 µl of Nafion 117 solution and 200 µL DI water. The linear sweep voltammetry (LSV) polarization curves were obtained at a scan rate of 10 mV/s. The uncompensated resistance (R_s_) and charge transfer resistance (R_ct_) were measured using the electrochemical impedance spectroscopic (EIS) techniques with a frequency range from 1 MHz to 1 Hz under the open-circuit potential, the Tafel slope was obtained by linear fitting, the plot of “log(i) versus η”. The electrochemical active surface area (ECSA) was evaluated through the double-layer capacitance (C_dl_) values measured from the CV curve within a non-faradic potential window at various scan rates of 10, 25, 50, and 100 mV/s.

## Results and discussions

### Morphology and structural characterizations

**Figure 2:**
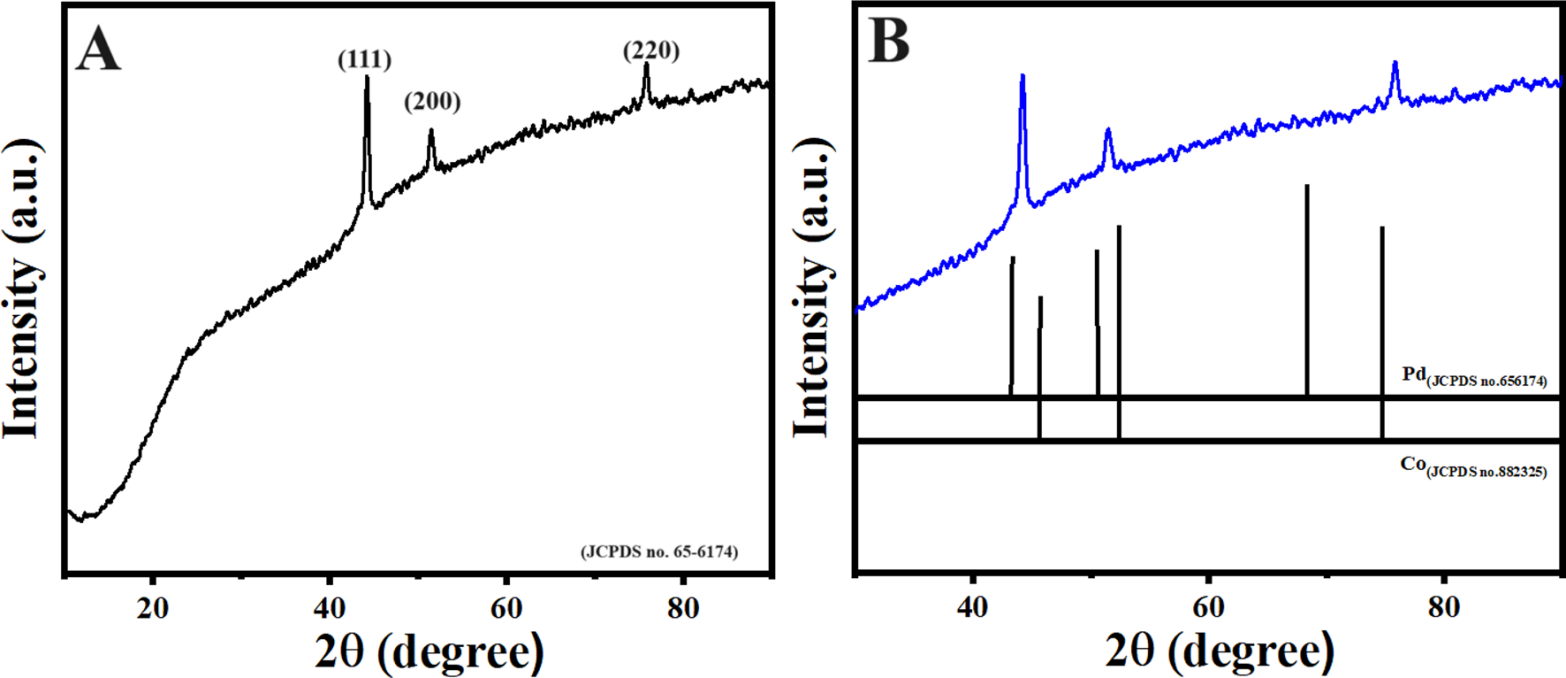
(**A**) XRD pattern for Pd-doped CoNPs@C electrode and (**B**) High magnification XRD pattern to show the peaks shift to higher 2θ values.

**Figure 3:**
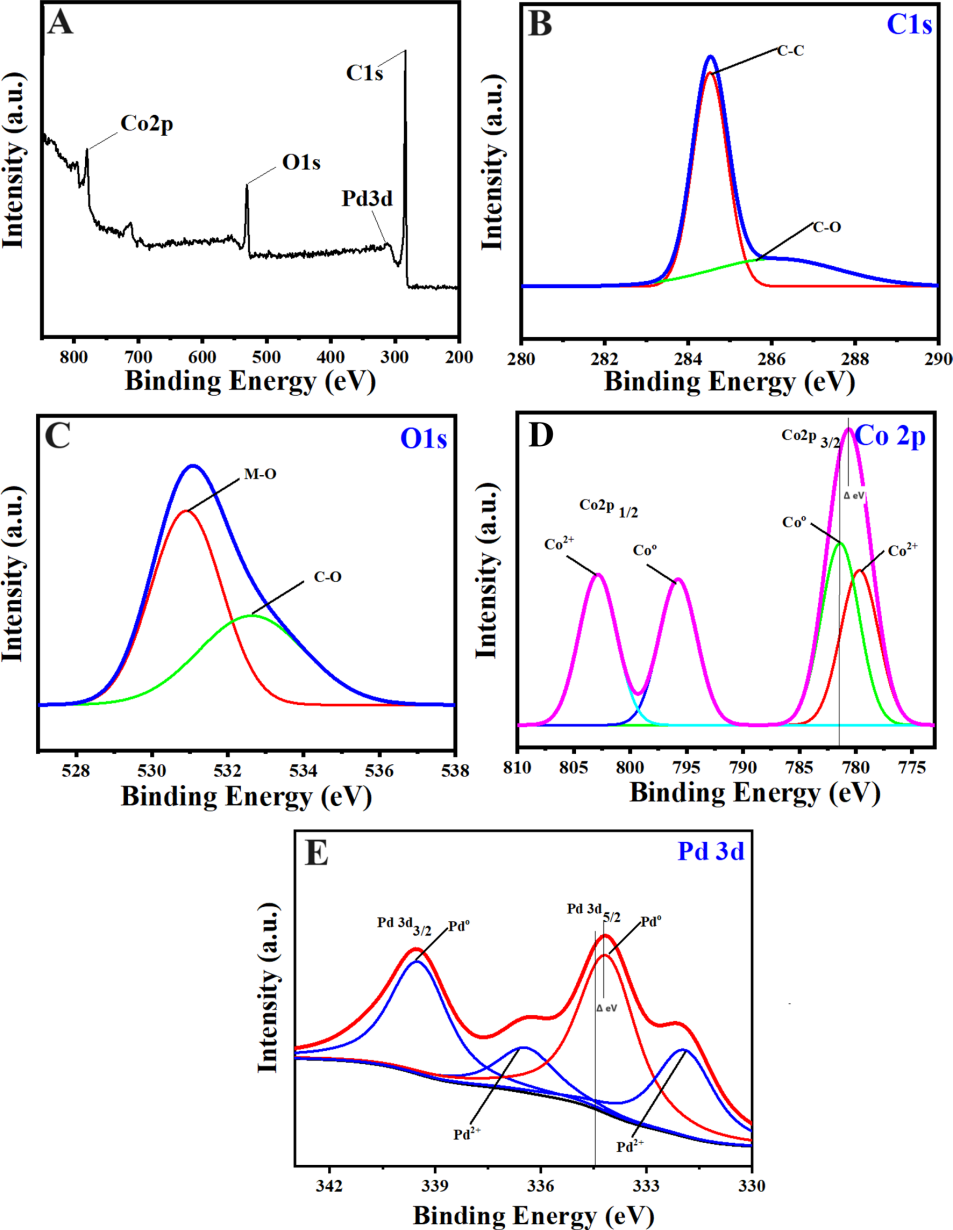
(**A**) XPS survey patterns, (**B**) C1s spectrum, (**C**) O1s spectrum, (**D**)Co2p spectrum and, (**E**) Pd3d spectrum for Pd-doped CoNPs@C electrode.

**Figure 4:**
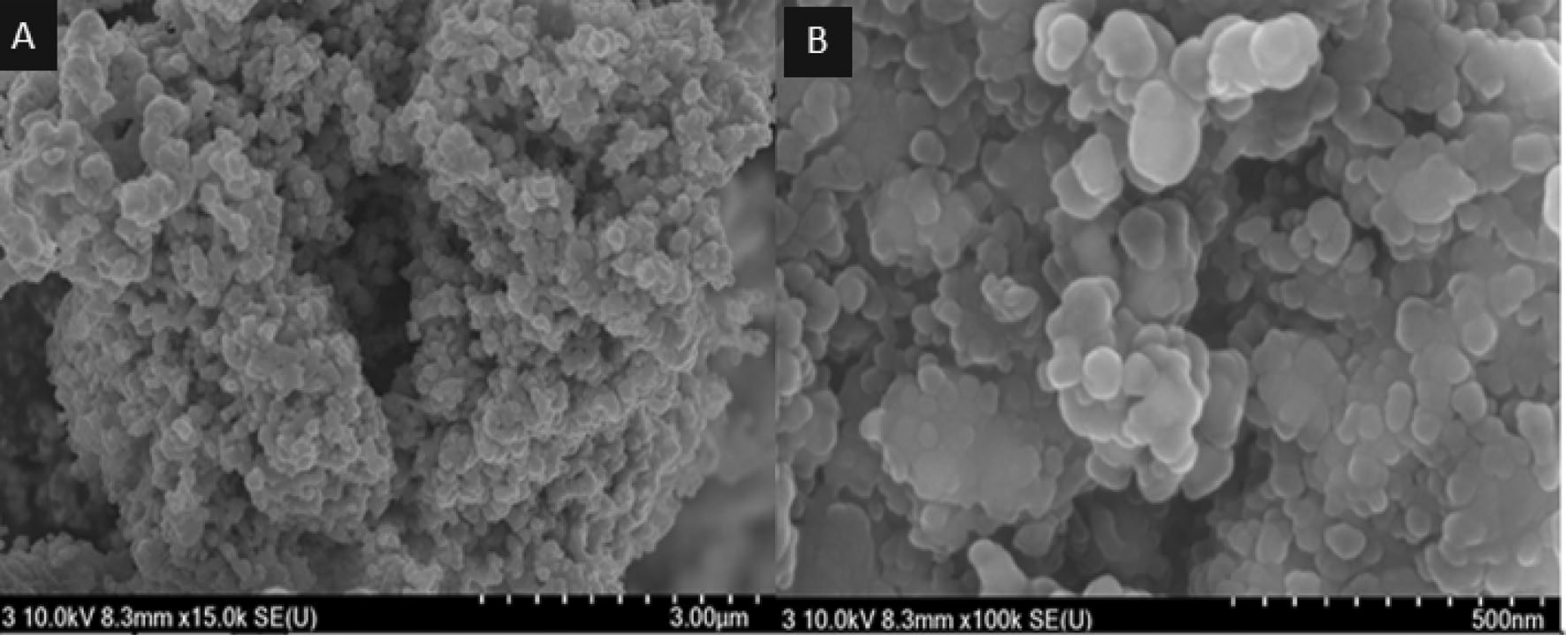
(**A**) Low and (**B**) High magnification SEM images for Pd-doped CoNPs@C electrode.

**Figure 5:**
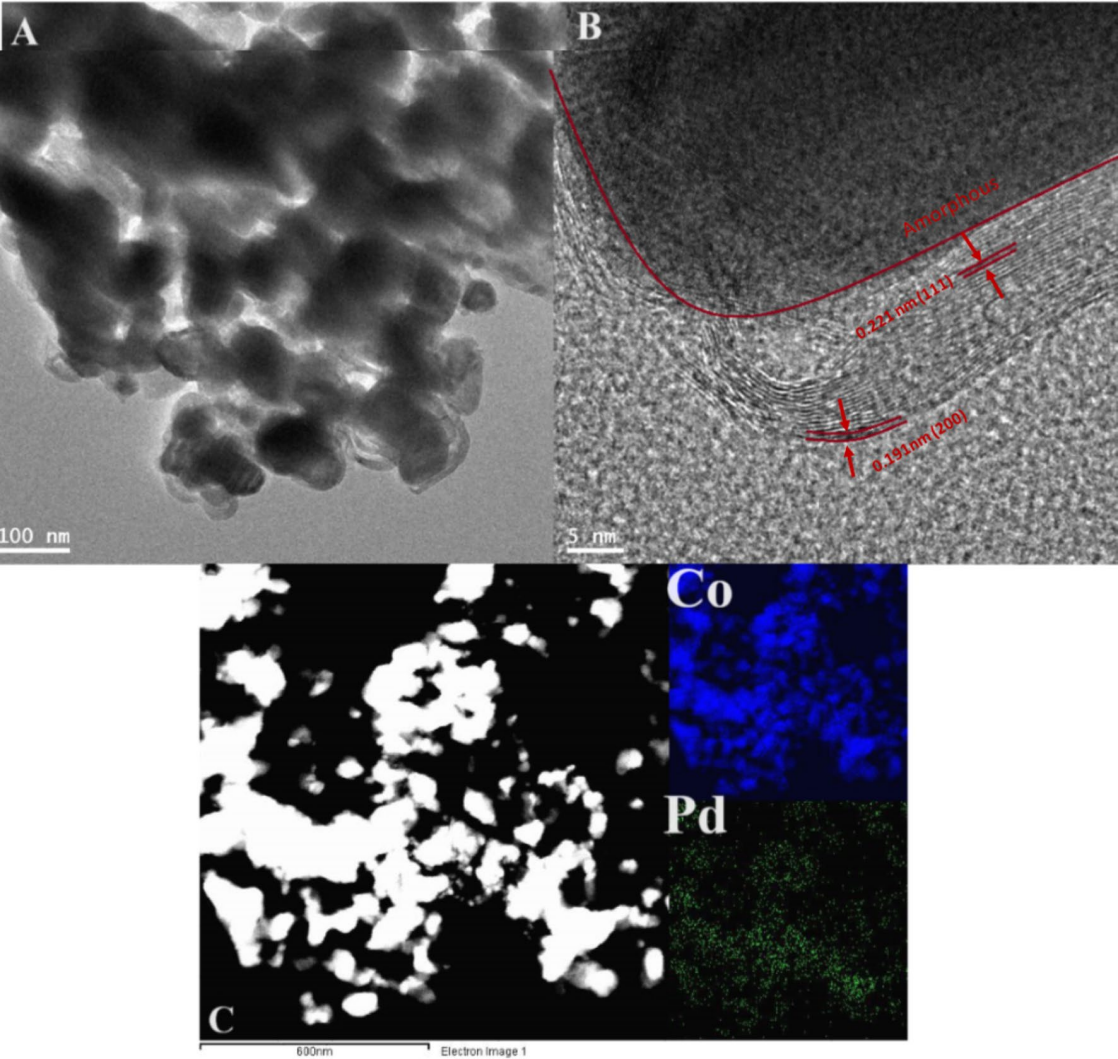
(**A**) TEM, (**B**) HR-TEM and (**C**) TEM–EDX image and corresponding element maps for Pd-doped CoNP @C electrode.

The X-ray diffraction measurements were carried out to determine the underlying crystal structure of the synthesized Pd-doped CoNPs@C electrode. As observed, the XRD pattern Fig. 2A shows three diffraction peaks located at 43.6°, 50.5° and 74.7° which are correspond well to the (111), (200) and (220) lattice planes of the face-centered cubic crystalline cobalt-palladium nanoparticles (JCPDS no. 65–6174), respectively. Meanwhile, compared with the standard patterns of Co (JCPDS: no. 88–2325) and Pd (JCPDS: no. 65–6174), the peak positions shifted to the large angles, which can be attributed to the insertion of Pd in the Co cubic structure (Fig. 2B)^8,43^. The X-ray photoelectron spectroscopy (XPS) was further employed to explore the surface chemistry of the synthesized Pd-doped CoNPs@C electrode. As observed, the full XPS spectrum Fig. 3A shows the major binding energies of Pd3d, Co2p, C1s and O1s at 320, 780, 286 and 531 eV, respectively which clarify the existence of Pd, Co, C and O in the synthesized Pd-doped CoNPs@C electrode. As can be observed in Fig. 3B, the high-resolution C1s spectrum can be deconvoluted into two obvious individual peaks of Sp2 carbon at 284.7 eV and C−OH at 286 eV^44^. On the other hand, the high-resolution O1s spectrum (Fig. 3C) can be deconvoluted into two peaks at 531.2 eV and 533 eV belong to metal oxide and C−OH, respectively^45^. However, the high resolution XPS spectra for Co2p (Fig. 3D) can be deconvoluted into 2p^1/2^ and 2p^3/2^ components, which confirm the presence of metallic and oxidic state of Co. As observed in Fig. 3D, a pair of peaks at 781.69 eV and 797.55 eV belong to metallic state Co, while a pair of peaks at 786.09 eV and 802.62 eV belong to the oxidic sate of Co. Similarly, as observed in high-resolution Pd3d spectra of synthesized Pd-doped CoNPs@C electrode presented in Fig. 3E can be deconvoluted into two components. A pair of peaks at 334.5 eV and 339.8 eV cab be assigned to the Pd 3d^5/2^ and 3d^3/2^ peaks of metallic Pd, while other pair of peaks at 335.2 eV and 340.4 eV are assigned to Pd 3d^5/2^ and 3d^3/2^ peaks of oxidic state of Pd. The existence of oxidic state of Pd/Co was probably attributed to the surface oxidation of metallic Co/Pd^46^. It is worth noting that both Pd3d and Co2p binding energies of the synthesized Pd-doped CoNPs@C electrode shift to the higher binding energies compared to their standard values, indicating the incorporation of Pd and Co in the synthesized Pd-doped CoNPs@C electrode. The binding energy shift in Pd3d and Co2p to the higher binding energies can be attributed to the existence of oxidic Co/Pd in PdCo, which is consistent with the literature^46^. However, inductively coupled plasma mass spectrometry (ICP-MS) result reveals that the Pd/Co molar ratio in the synthesized Pd-doped CoNPs@C electrode was 8:2. The morphology and structure of the synthesized Pd-doped CoNPs@C electrode were investigated by SEM and TEM techniques. As observed in the SAM image (Fig. 4A) the synthesized Pd-doped CoNPs@C electrode maintains quite spherical structure with cluster of uniformly distributed particles. Moreover, the average particles size was in the range of 50–100 nm (Fig. 4B). On the other hand, as shown in the TEM image (Fig. 5A) the metallic nanoparticles enveloped by the graphite shell. However, the high magnification TEM image (Fig. 5B) stated that the synthesized Pd-doped CoNPs@C electrode comprises of both crystalline and amorphous parts (marked by solid line). Further, close observation shows more than one lattice spacing of 0.221 and 0.191 nm can be attributed to the (111) and (200) planes of the Pd-Co alloying, respectively. This observation simultaneously supports the XRD results (Fig. 2A. What’s more the elemental mapping on the randomly selected area shows that Pd and Co are uniformly distributed over the whole nanoparticles (Fig. 5C). However, Co is dominant (94.1%) followed by C (4.61%) and Pd (1.29%).

### Electrocatalytic performance

#### Electrocatalytic OER performance in 1.0 M KOH aqueous solution

**Figure 6:**
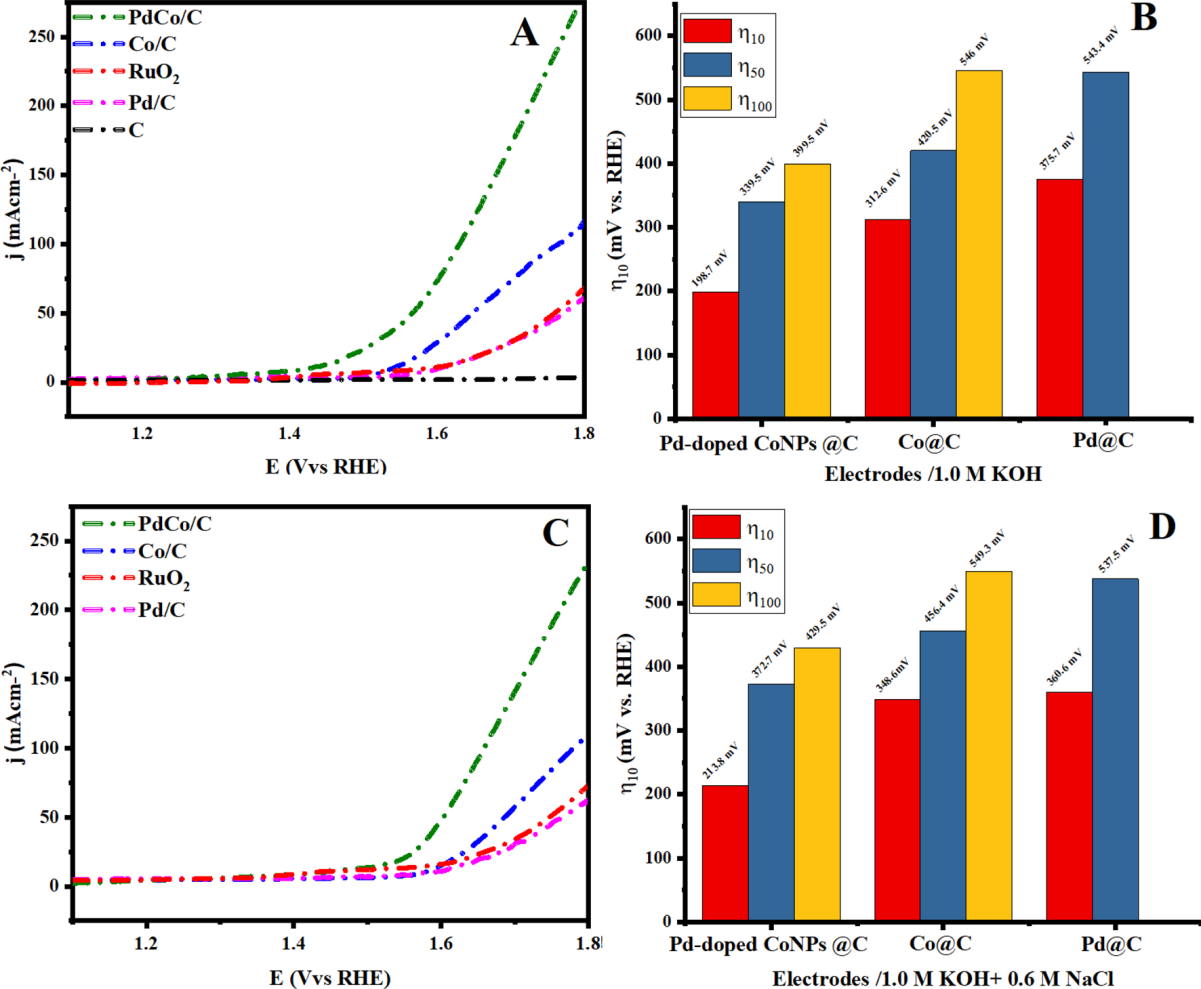
(**A**) and (**B**) OER polarization curve and overpotential to reach the current density of 10, 50 and 100 mA/cm^2^ in 1 M KOH at a scan rate of 10 mV/s and (**C**) and (**D**) OER polarization curve and overpotential to reach the current density of 10, 50 and 100 mA/cm^2^ for prepared Pd@C, Co@C, pristine C and Pd-doped CoNPs@C electrode in 1.0 M KOH + 0.6 M NaCl at a scan rate of 10 mV/s, along with RuO_2_ for comparison.

To assess the efficiency of the developed electrocatalyst, the OER electrocatalytic performance for prepared Pd-doped CoNPs@C, Pd@C, Co@C, and pristine C electrodes, along with RuO_2_ electrode was investigated by LSV using the conventional three-electrode setup in 1.0 M KOH aqueous solution at a scan rate of 10 mV/s. As seen in Fig. 6A, the Pd-doped CoNPs@C electrode exhibit a small onset potential with a high current response when compared with the Pd@C, Co@C, and pristine C electrode. Importantly, as seen, the Pd-doped CoNPs@C electrode had a very low overpotential of 198.7 mV to deliver the current density of 10 mA/cm^2^, which is comparable to that of commercial RuO_2_ electrode under the same conditions^47^. Figure 6B also shows that the Pd-doped CoNPs@C electrode achieved the current density of 50 and 100 mA/cm^2^ at the overpotential of 339.5 and 399.5 mV, respectively, while the required overpotential values to afford ƞ_10, and_ ƞ_50,_ current density for Pd@C (ƞ_10_ = 375.7 mV, ƞ_50_ = 543.4 mV), and Co@C (ƞ_10_ = 312.6 mV, ƞ_50_ = 420.5 mV) was much higher than Pd-doped CoNPs@C electrode.

**Figure 7:**
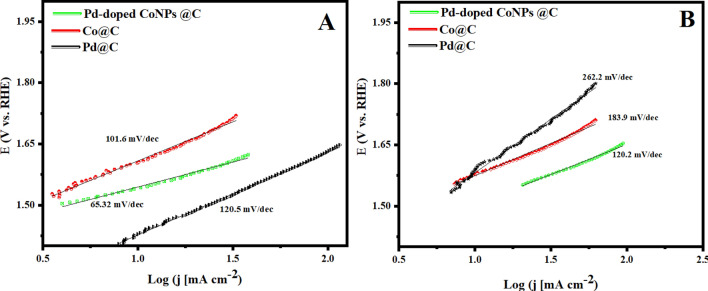
Tafel slope for prepared Pd@C, Co@C and Pd-doped CoNPs@C electrode (**A**) in 1 M KOH and (**B**) in 1.0 M KOH + 0.6 M NaCl.

To further validate our findings, we deliberately study the kinetic behavior of Pd-doped CoNPs@C, Pd@C, and Co@C electrodes towards the OER reaction at the electrode–electrolyte interface using the Tafel plot. As seen in Fig. 7A, the corresponding Tafel slope for the Pd-doped CoNPs@C electrode was the smallest (65.32 mv/dec.) when compared with Pd@C (120.5 mv/dec.), and Co@C electrode (101.6 mV/dec.). The results indicate that the Pd-doped CoNPs@C electrode have faster reaction kinetics and superior OER activity when compared to the Pd@C and Co@C electrode.

#### Electrocatalytic OER performance in alkaline seawater medium

After demonstrating such a good electrocatalytic performance of Pd-doped CoNPs @C electrode in an alkaline medium, it is worth performing the same electrochemical analyses in an alkaline seawater medium. Therefore, the electrocatalytic OER performance of the Pd-doped CoNPs@C electrode was evaluated in 1 M KOH + 0.6 M NaCl. As shown in Fig. 6C Pd-doped CoNPs@C electrode exhibit slight activity decay in 1 M KOH + 0.6 M NaCl, but as expected, Pd-doped CoNPs@C electrode demonstrate reasonably good OER activity among other formulations with a low overpotential of ≈213 mV to deliver a current density of 10 mA/cm^2^, followed by Co@C (≈ 348 mV) and Pd@C (≈ 657 mV). Nevertheless, it is worthwhile to mention that to deliver a current density of 100 mA/cm^2^, the overpotential (≈ 429.5 mV) required for Pd-doped CoNPs@C electrode is lower than that to initiate hypochlorite formation (≥ 490 mV) as shown in Fig. 6D. The corresponding Tafel slope (Fig. 7B) for all the formulations shows significant increases compared to the values obtained under the alkaline conditions. The existence of Cl^−^ may contribute to its OER reaction kinetics degradation, as it is easily adsorbed and block the active sites of the metal-based electrocatalysts system^48^.

**Figure 8:**
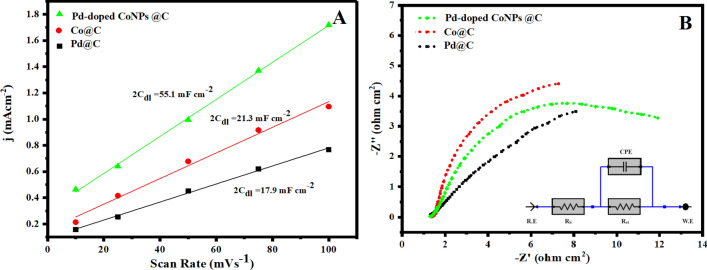
(**A**) Electrochemical surface areas (ECSA) and (**B**) Nyquist plot (inset) corresponding equivalent circuit diagram for prepared Pd@C, Co@C and Pd-doped CoNPs@C electrode.

**Figure 9:**
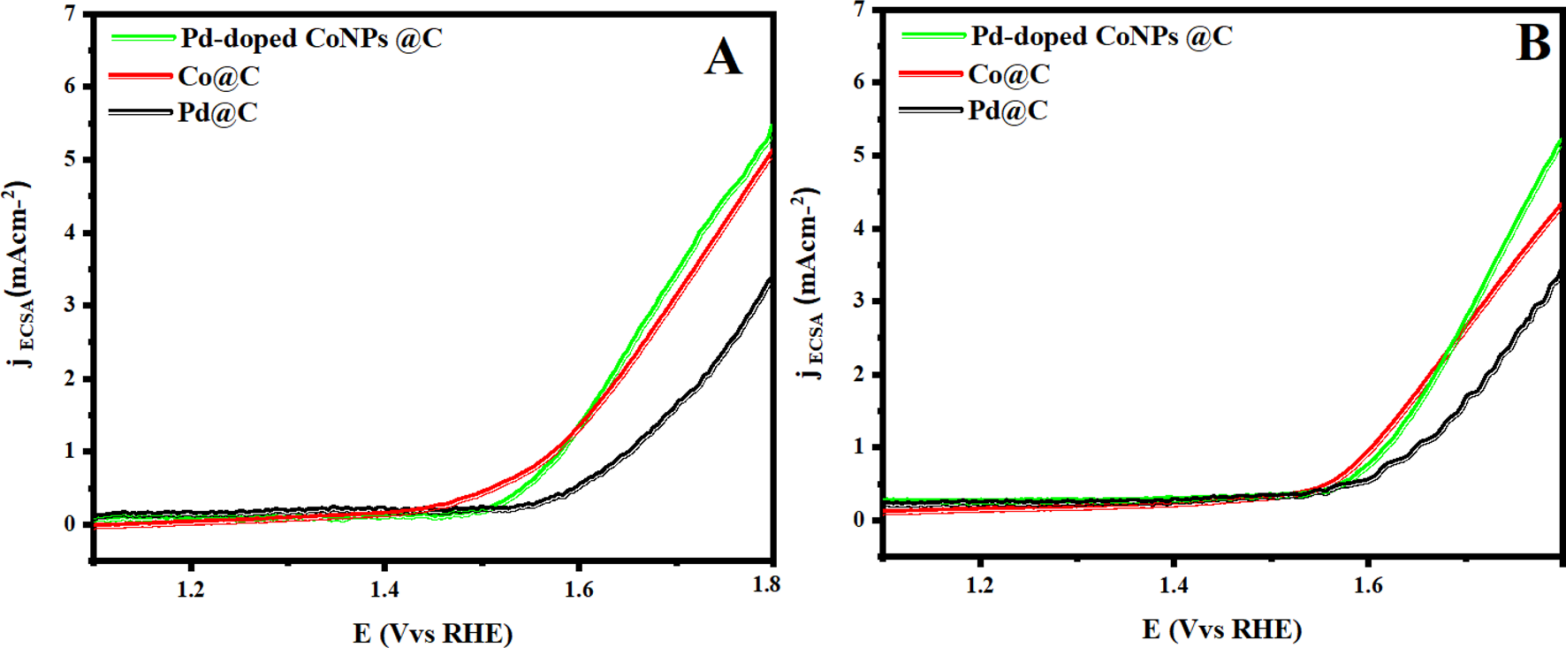
(**A**) OER polarization curve normalized to the electrochemical surface area in 1 M KOH at a scan rate of 10 mV/s and (**B**) OER polarization curve normalized with electrochemical surface area 1.0 M KOH + 0.6 M NaCl at a scan rate of 10 mV/s.

**Figure 10:**
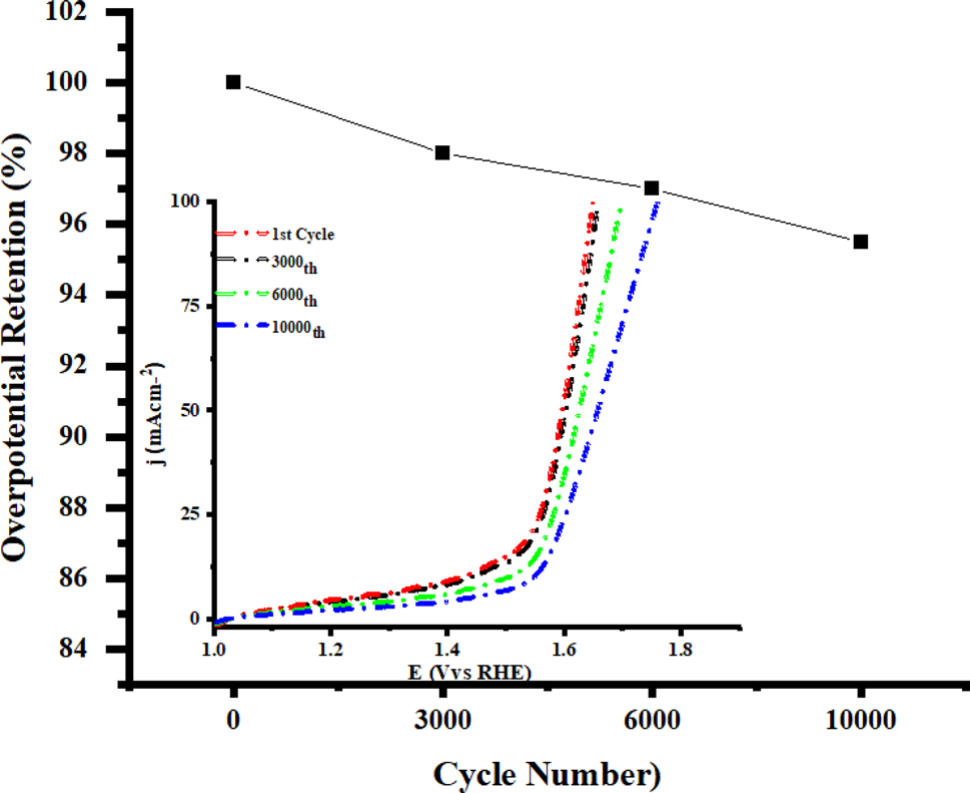
Overpotential retention as a function of LSV cycle number and (inset) corresponding LSV cycles for prepared Pd-doped CoNPs@C electrode.

To figure out the enhanced OER activity of Pd-doped CoNPs@C electrode over the Co@C and Pd@C electrode, ECSA and EIS techniques have been used. First, ESCA of Pd-doped CoNPs@C, Co@C, and Pd@C electrode was explored using the CV test as the ESCA is equal to the C_dl_ that could be obtained from the CV curves. These CV curves were measured in the non-faradaic region at various scan rates of 10, 25, 50, 75 and 100 mV/s vs RHE (Supporting information, Figure S1). As seen in Fig. 8A, the Pd-doped CoNPs@C electrode deliver the highest C_dl_ value (55.1 mF/cm^2^) compared to Pd@C electrode (17.9 mF/cm^2^) and Co@C electrode (21.9 mF/cm^2^). The higher C_dl_ value delivered by Pd-doped CoNPs@C electrode shows the more accessible active sites for OER. Further, EIS was recorded to examine the inherent conductivity of Pd-doped CoNPs @C electrode and all other reference electrodes. As seen in Fig. 8B, Pd-doped CoNPs@C electrode showed smaller semicircles loop diameter compared to the Pd@C electrode and Co@C electrodes. A Randle circuit model can be used to deduce the EIS data. Based on the equivalent circuit (inset Fig. 8B) Pd-doped CoNPs@C electrode showed much lower Rct (12.34 Ω cm^2^), suggesting that electron transfer between electrode and electrolyte is very fast. To understand the factual contribution, the OER current densities of Pd-doped CoNPs@C electrode, Pd@C and Co@C electrode have been normalized to the corresponding C_dl_, as shown in Fig. 9. It is worth observing that Pd-doped CoNPs@C electrode exhibit the highest C_dl_ normalized OER performance especially compared to all other references as can be seen in Fig. 9A,B. The cyclic stability of OER electrocatalysts is also a critical challenge for practical applications. Therefore, the LSV check was executed at 100 mV/s for 10,000 consecutive cycles. As can be seen, Pd-doped CoNPs@C electrode is quite stable but gradual shift in the potential after 3000 LSV cycles is attributed to the bubbles accumulation at the active area of the electrode (Fig. 10). However, only 6% overpotential was increased to maintain the same current density after 10,000 cycles (inset Fig. 10).

However, based on the above studies, the mechanism is established to underline the improved catalytic activity of Pd-doped CoNPs @C electrode toward OER can be attributed to the following unique features.

1. The structural change due to doping of Pd nanoparticles can improve intrinsic catalytic activity, reduce activation energy and enhance the adsorption of OH^−^ and OOH^−^ intermediates toward OER, which can be realistically considered through the synergistic effect of individual elements and the rich interfaces (i.e. Pd–Co–C)^49^. In addition, integrating the inherent conductive characteristic of graphitic carbon around PdCo nanoparticles can also promote potent charge transfer between Pd-doped CoNPs@C electrode and electrolyte, which is proven by the low charge transfer resistance^50^.
2. In accordance with ECSA results, Pd nanoparticle doping has significantly enhanced the Co/C active sites, which can reasonably be considered toward the OER responses. What’s more, from the XPS measurements (Fig. 3D,E), it can be claimed that the weak oxophilicity of Pd-doped CoNPs@C electrode is also responsible for the improved OER activity^51^.
3. Besides the above, EIS results further confirm that the doping of Pd nanoparticles gives rise to low internal resistance and fast charge transfer for a low onset potential and high OER kinetics. In addition, numerous Pd-doped Co nanoparticles are assembled on the graphitic carbonaceous nanostructure, which can offer a pathway for fast electron transport from the electrode and electrolyte^52^.
4. Finally, the distinctive morphology makes Pd-doped CoNPs@C electrode possesses excellent OER activity and durability.

## Conclusion

We propose Pd-doped Co nanoparticles encapsulated in graphitic shell (Pd-doped CoNPs@C electrode), as an effective and cost efficient electrocatalyst for OER in water electrolysis, more specifically for alkaline seawater electrolysis. The Pd-doped CoNPs@C electrode shows reasonably high activity and selectivity over chlorine evolution reaction (CER) under alkaline pH conditions for seawater oxidation. The synergistic effect of individual elements and the rich interfaces (i.e., Pd–Co–C) significantly contributed to the increased electrochemical active surface area and reasonable improved electrode performance (i.e., low internal resistance, low onset potential, fast charge transfer and high OER kinetics) were clearly observed. In conclusion, this study provides quite sufficient experimental details for research and development of efficient and cost-effective electrocatalysts for seawater oxidation.

### Supplementary Information

Supplementary Figure S1.

## Data Availability

All data generated or analyzed during this study are included in this published article.
