# Supplementary material for: Nanoengineered, Pd-doped Co@C nanoparticles as an effective electrocatalyst for OER in alkaline seawater electrolysis
Source: Sci Rep. 2023 Nov 27;13:20866. doi: 10.1038/s41598-023-46292-9 (PMC10682028; doi:10.1038/s41598-023-46292-9)
Supplement: Supplementary file 1 — Supplementary Figure S1. [file 41598_2023_46292_MOESM1_ESM.docx]

**Supporting information**

**Nanoengineered, Pd-doped Co@C Nanoparticles as an effective Electrocatalyst for OER in Alkaline Seawater Electrolysis**

**Zafar Khan Ghouri^1,2*^, David James Hughes^1^, Khalid Ahmed^3^, Khaled Elsaid^4^,**

**Mohamed Mahmoud Nasef^2,5^, Ahmed Badreldin^4^, and Ahmed Abdel-Wahab^4*^**

# ^1^School of Computing, Engineering and Digital Technologies, Teesside University, Middlesbrough, Tees Valley, TS1 3BX, UK

^2^Center of Hydrogen Energy, Institute of Future Energy, Universiti Teknologi Malaysia, Jalan Sultan Yahya Petra, 54100 Kuala Lumpur, Malaysia

^3^International Center for Chemical and Biological Sciences, HEJ Research Institute of Chemistry, University of Karachi, Karachi-75270, Pakistan

^4^Chemical Engineering Program, Texas A&M University at Qatar, P.O. 23874, Doha, Qatar

^5^Malaysia-Japan International Institute of Technology, Universiti Teknologi Malaysia, Jalan Sultan Yahya Petra, 54100 Kuala Lumpur, Malaysia


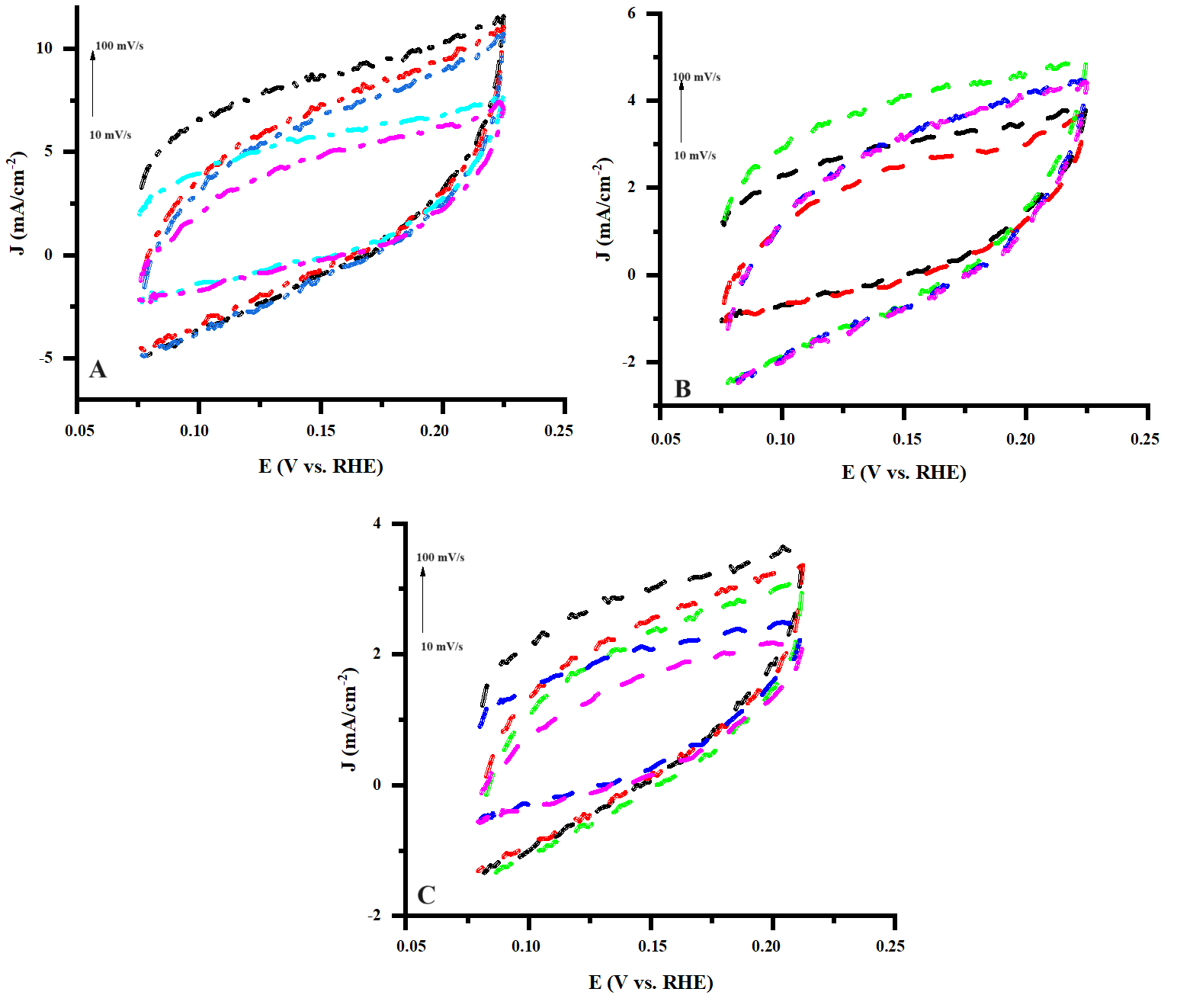


Figure.S1. CV curves for prepared (A) Pd-doped CoNPs@C (B) Co@C and (C) Pd@C electrode at different scan rates from 10 to 100 mV/s
